# Treatment patterns and healthcare resource utilization for triple negative breast cancer in the Brazilian private healthcare system: a database study

**DOI:** 10.1038/s41598-023-43131-9

**Published:** 2023-09-22

**Authors:** Maria Amelia Carlos Souto Maior Borba, Paula de Mendonça Batista, Milena Falcão Almeida, Maria Aparecida do Carmo Rego, Fernando Brandão Serra, Julio Cesar Barbour Oliveira, Karina Nakajima, Guilherme Silva Julian, Gilberto Amorim

**Affiliations:** 1 MSD Brazil, Avenida Chucri Zaidan, 296-11º Andar, Edif. Torre Z Vila Cordeiro, São Paulo, SP CEP: 04583-110 Brazil; 2 IQVIA Brazil, São Paulo, Brazil; 3 Oncologia D’OR, Rio de Janeiro, Brazil

**Keywords:** Breast cancer, Breast cancer, Health care economics

## Abstract

In Brazil, data on the management of triple negative breast cancer (TNBC) as well as the burden of the disease in terms of health care resources utilization (HCRU) are scarce. To characterize the treatment patterns and HCRU associated with the management of Brazilian TNBC patients from the perspective of the private healthcare setting. Patients with at least one claim related to ICD-10 C50 from January 2012 until December 2017, and at least one claim for breast cancer treatment were assessed from a private claims database and classified as early and locally advanced, or metastatic. All patients with hormone and/or targeted therapy were excluded. Three thousand and four patients were identified, of which 82.8% were diagnosed in early and locally advanced stages. For early and locally advanced TNBC patients, 75.3% were treated in an adjuvant setting, mainly with anthracycline regimes. For mTNBC patients, bevacizumab regimens were the main treatment prescribed. More than 48% of mTNBC patients were switched to a second line of treatment. HCRU was higher for mTNBC patients when compared to early and locally advanced patients, with higher costs for metastatic disease management. The treatment setting has little influence on the HCRU pattern or the cost of disease management. The highest burden of disease was observed for metastatic management.

## Introduction

Triple negative breast cancer (TNBC) is characterized by the lack of expression of the estrogen receptor, progesterone receptor and human epidermal growth receptor 2 (HER2)^1^. TNBC accounts for 15–20% of all breast cancer (BC) diagnosed worldwide^2–4^ and is associated with a worse prognosis, a higher rate of distant metastasis, and shorter survival after recurrence^5,6^.

Due to the absence of targetable receptors, standard cytotoxic chemotherapy remains the predominant treatment option for patients with TNBC, in both neoadjuvant and adjuvant settings^7^. Anthracycline and taxane-based chemotherapy are the main treatments in clinical practice, especially during early stages^5^. In these cases, both adjuvant therapy (AT) and neoadjuvant therapy (NAT) have similar overall survival rates^8,9^, hence, the treatment setting decision is made according to the clinical-histopathological staging characteristics at diagnosis^10^. Nevertheless, NAT is still the mainstay strategy as it allows tumor downstaging (aiming at a more conservative surgery), and better monitoring of chemotherapy-resistant tumors^11^. Despite chemotherapy, one in three TNBC patients will develop tumor recurrence, mainly within the first three years of diagnosis^7^. The 5-year overall survival rates for localized, regional, and metastatic TNBC patients are 91%, 65%, and 11%, respectively^7^.

In Brazil, TNBC is most detected in stage III, with lymphocytic infiltration, multifocality, and tumor size > 2 cm at diagnosis^12,13^. Compared to other BC subtypes, a higher proportion of patients with TNBC undergo radical surgery and chemotherapy. Most patients treated with NAT receive anthracycline followed by taxane regimens^13,14^. Overall survival of Brazilian TNBC patients tends to be lower than that observed worldwide, mainly due to late diagnosis and difficult access to healthcare services^12^.

TNBC also has a great impact on healthcare resource utilization (HCRU), with higher hospital admissions and emergency room (ER) visits compared to patients with other BC subtypes^15–17^. However, data on the economic burden of TNBC remains scarce, especially in Brazil. Expenditures for the private healthcare system are high, totaling 77% of all oncology expenditures in Brazil^18^. The lack of data on treatment patterns, HCRU and costs of TNBC treatment and management are limited, hinders the development of accurate pharmacoeconomic studies, policy planning and private system budget allocations^19^.

Therefore, the aim of this study was to describe real world data on HCRU and treatment patterns of TNBC in the Brazilian healthcare system.

## Methods

### Study design and population

This was a database study to assess TNBC patient management from the perspective of the private healthcare system. The database used was the Orizon, which comprised 13 million beneficiaries from the private health sector. In December 2017, the total private health system had over 47 million beneficiaries^20^.

Patients 18 years or older, with at least one BC claim (ICD-10 C50) from January 2012 until December 2017, and at least one claim for a BC treatment usually prescribed for TNBC patients (inclusion molecules) were selected. Patients with targeted and/or hormone therapies (exclusion molecules) or with any BC treatment or ICD-10 C50 claims in 2011 were excluded due to low quality of data. Period also considers the 7th edition of the staging guideline for breast cancer^21^. The inclusion and exclusion molecules are listed in Supplementary Table 1.

The stage at index date (considered as the date of the first ICD-10 claim reported) was inferred based on the identification of specific treatments and procedures aimed at metastatic disease management. The algorithm used to classify whether the patient had metastatic (stage IV) or early and locally advanced (stages 0–III) TNBC was adapted from previous claims database studies^22,23^. Briefly, patients with ≥ 2 claims of metastatic ICD-10 codes (C76 to C80, except for C77.3—secondary and non-specified malignant tumor of axillary and upper limbs lymph nodes) or metastatic related treatments and/or procedures (Supplementary Table 2), with at least 15 days apart, reported between 1 month before and 6 months after the index date were considered to have mTNBC. Patients who did not present these criteria were considered to have early and locally advanced TNBC.

### Treatment pattern

The treatment pattern was assessed following the line of treatment (LOT) definition described elsewhere^24^. The date of the first claim for any of the pre-defined drugs was considered as the treatment initiation. A treatment switch was considered when claim of a different drug from the current treatment regimen was reported after 60 days or more, or when a drug claim was reported after an interval of at least 120 days. For early and locally advanced TNBC patients, if a new drug was identified within a window of 45 days it was considered as a sequential treatment. If a subsequent regimen was identified after the 45-day treatment period, it was considered as a treatment for progressive disease, thus the term used forward was LOT.

### Healthcare resource utilization

A longitudinal assessment was performed considering both inpatient and outpatient settings to compose HCRU and costs. For HCRU we assessed outpatient care separately, which comprised all outpatient visits (office visits), procedures (all ancillary care procedures), and ER visits. All costs associated with outpatient visits, procedures and ER visits were grouped to compose the outpatient costs. The medications were described in both outpatient and inpatient claims within Orizon database; however, the medication costs attributed to each setting could not be evaluated due to database limitations. Thus, the medication costs were not assessed. For inpatient care, the number of admissions, length of stay, and costs were assessed. Costs in Brazilian Reais (BRL) were adjusted to Mar/2023 using the “Citizen Calculator” of the Central Bank of Brazil, based on the IPCA (IBGE) index (average inflation index of 1.5769934 for 2012–2017). Then, all costs were converted to US dollars (USD) using an average exchange rate (1 BRL = 0.1968581 USD on March 31, 2023). All breast-related surgery costs were included in the inpatient setting. The total cost for BC management is the sum of the outpatient and inpatient costs.

### Data source

Data was extracted from the Orizon database, an administrative claims database that covers several private health maintenance organizations (HMOs) in Brazil. This private database included the patients’ demographic information, procedures, materials, medications, and costs. All patient information provided was de-identified. The dataset used is from a private source and there is no public link for its access since its content is understood as intellectual property. If a reader wants to have access to the data or consult any specific point related to it must contact the corresponding author by email.

### Data analysis

The treatment pattern was described and summarized as frequency. Treatment duration was presented as the median (IQR) and calculated as the total number of months since the first treatment initiation (first treatment claim) and the end of the respective treatment (considered as the last treatment claim). The time to next treatment (TTNT) was calculated as the total number of months elapsed since the first treatment claim (treatment initiation) until the next treatment initiation (first claim of the next treatment line).

The HCRU and associated costs were presented as per patient per month (PPPM). The PPPM metric was calculated as the mean event and associated costs per patient during a month. For HCRU, a Poisson distribution was considered to estimate the 95% confidence intervals (CI). For cost measures and procedures, a gamma distribution was considered to estimate 95% CI.

For HCRU and costs we only considered the treatment duration period. Periods between treatments were not considered to avoid the inclusion of resources not related to the BC management.

### Ethic approval and consent to participation

This study was performed in accordance with Good Pharmaepidemiology Practice (GPP) and Brazil regulations. This was a retrospective observational study and did not impose any form of intervention to the patients. The assessment and treatment of the cases was based on an anonymized private database, to protect the confidentiality and privacy of patients included in the database. Therefore, a formal approval from Ethics Committee and Consent form is not required accordingly to Resolution 510/2016 Art.1.

## Results

### Patient characteristics

**Figure 1:**
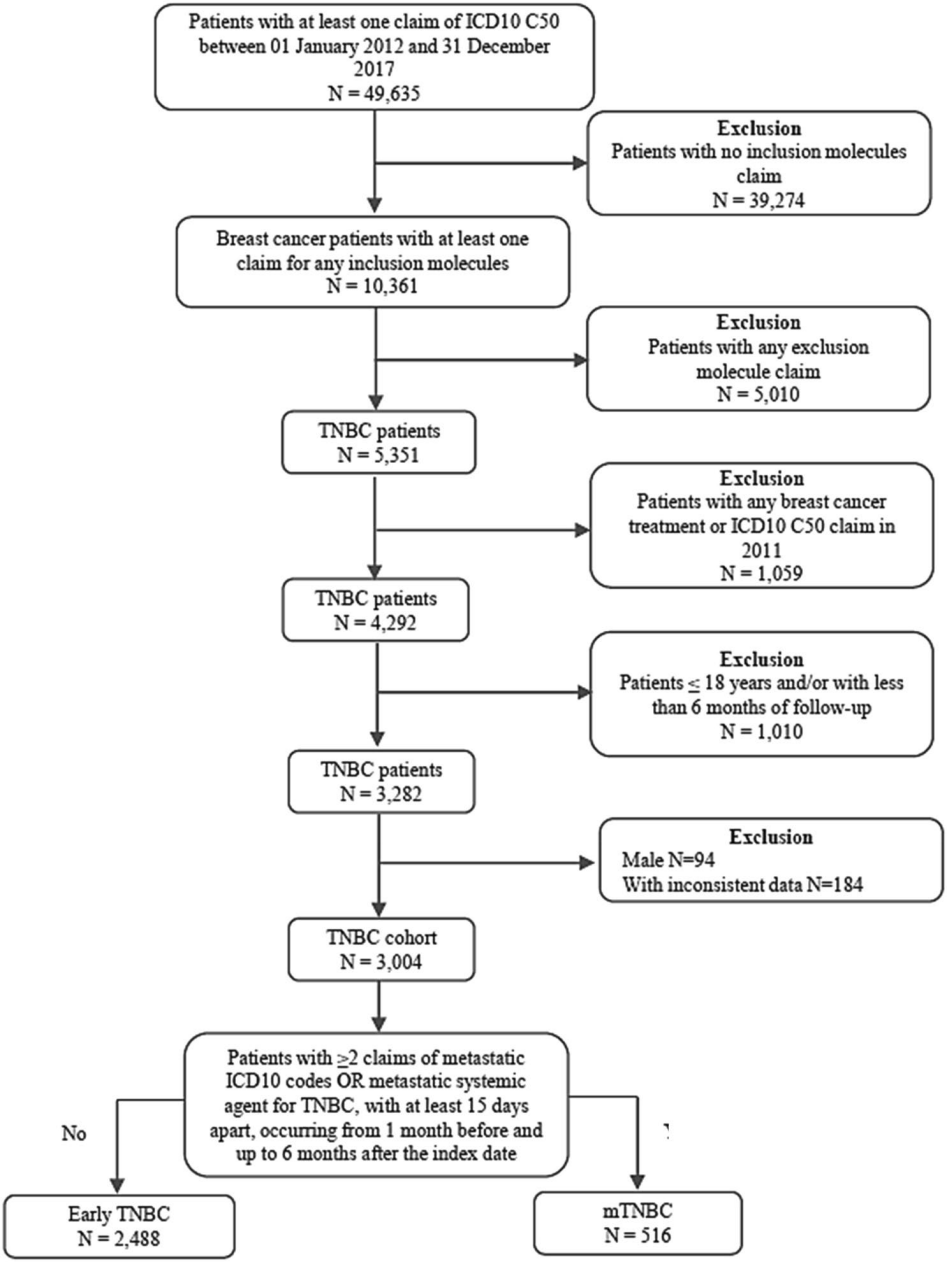
Patient selection. TNBC: triple negative breast cancer.

From the 49,635 BC patients, 3004 (6%) were TNBC. The tumor stage classification revealed 2488 (82.8%) patients with early and locally advanced TNBC and 516 (17.2%) with mTNBC (Fig. 1). The demographic characteristics are reported in supplementary Table 3. The median age at the index date was 48 years, and among patients who had this information available, the majority were between 30 and 59 years old (38.9%). The median follow-up period, in years, was 2.25 for metastatic and 1.91 for early and locally advanced TNBC patients. Overall, 68.5% of TNBC patients were diagnosed in the Southeastern region. Breast surgery was performed in 41.6% and 37.4% of early and locally advanced, and metastatic patients, respectively. Only 5.9% of all TNBC patients had a claim reported for radiotherapy.

### Treatment pattern

**Figure 2:**
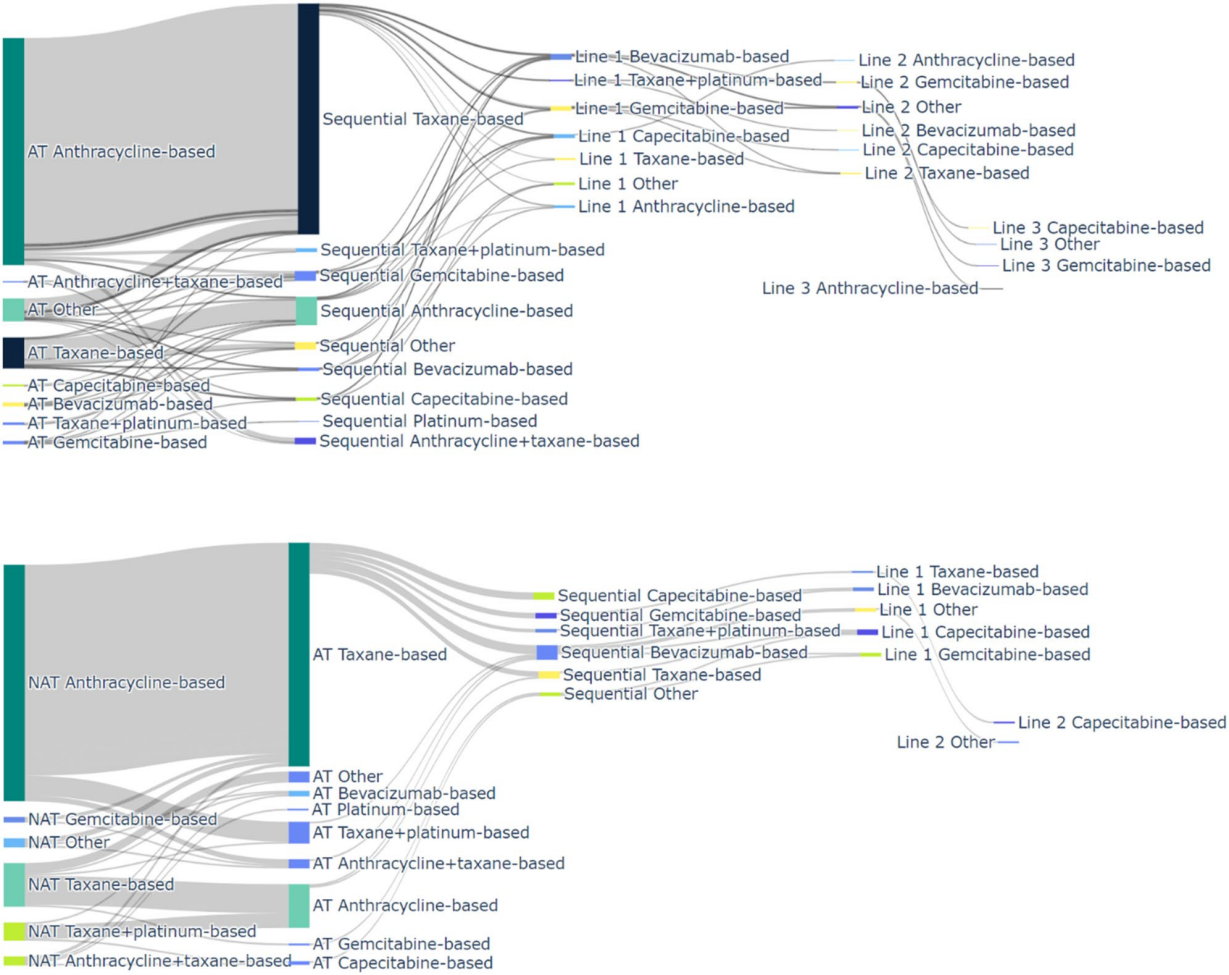
Sankey diagram of treatment patterns for early and locally advanced TNBC, in AT and NAT/AT settings.

For early and locally advanced TNBC treatment patterns and HCRU, we only assessed patients with a breast surgery claim (N = 1034). In this group, 75.3% of patients received AT; 7.5% received only NAT, and 17.1% received both NAT and AT. Sequential treatment was prescribed for 57.3% and 13.0% of patients treated in AT and NAT/AT, respectively. The treatment regimens varied according to treatment setting as shown in supplementary Table 4. In the NAT group, most patients received a combination of anthracycline and taxane chemotherapy (doxorubicin, cyclophosphamide, and paclitaxel/docetaxel—AC-T). In the AT group, the combinations prescribed the most were anthracycline regimens (mainly doxorubicin plus cyclophosphamide—AC). As observed in the Sankey diagram (Fig. 2), most AT patients with anthracycline regimens received taxane (especially paclitaxel) as a sequential chemotherapy. Conversely, patients treated with adjuvant taxane regimens (docetaxel plus cyclophosphamide or docetaxel/paclitaxel alone) received anthracycline (mainly AC) as a sequential chemotherapy. Most patients in the NAT/AT group received anthracycline regimens (AC or doxorubicin alone) in the neoadjuvant setting followed by taxane regimens (paclitaxel/docetaxel alone) in the adjuvant setting (Fig. 2).

For mTNBC patients, 39.9% of those with a breast-related surgery claim received NAT (77 patients out of 193). In this group 74.4% of patients were prescribed anthracycline-based regimens (AC or doxorubicin alone). Additionally, 98.6% of mTNBC patients had a LOT1, with 48.4% switching to a subsequent LOT. For LOT1, bevacizumab regimens accounted for 21.6% of all prescriptions, mainly bevacizumab in combination with paclitaxel. As for the LOT2, 34.4% received taxane regimens (mainly paclitaxel alone), and 18.4% received gemcitabine regimens (gemcitabine alone or in combination with cisplatin). Among those treated in LOT2, only 12.6% went to LOT3, 21.5% was treated with capecitabine and 15.4% with gemcitabine (supplementary Table 5).

For early and locally advanced TNBC patients, the median time from the index date until NAT initiation was 0.3 months (IQI 0.00–0.90) and from the index date until surgery was 0.7 months (IQI 0.00–5.60). For AT group the median time from surgery until AT initiation was 1.4 months (IQI 0.70–2.10). For mTNBC patients, the median time from the index date until LOT1 initiation was 1.7 months (IQI 0.17–3.33). Considering TTNT, the median time from LOT1 until LOT2 was 0.93 months (IQI 0.70–3.21); and from LOT2 until LOT3 was 1.4 months (IQI 0.70–3.73) (Table 1).

**Table 1:** Time to treatment initiation and time to next treatment in months for early and locally advanced, and metastatic TNBC patients.

	n	Mean ± SD (months)	Median (months)	IQI
Early and locally advanced TNBC				
Time from index date until NAT	255	0.66 ± 1.02	0.3	0.00–0.90
Time from index date until surgery	1034	4.45 ± 9.97	0.7	0.00–5.60
Time from surgery until AT	956	0.98 ± 10.20	1.4	0.70–2.10
mTNBC				
Time from index date until LOT1	509	3.50 ± 7.71	1.7	0.17–3.33
LOT1 treatment duration		3.91 ± 4.67	2.33	1.40–4.19
Time from LOT1 until LOT2	250	3.20 ± 5.53	0.93	0.70–3.21
LOT2 treatment duration		2.91 ± 2.44	2.8	1.63–3.26
Time from LOT2 until LOT3	65	3.37 ± 5.51	1.4	0.70–3.73
LOT3 treatment duration		2.65 ± 2.54	1.86	1.17–3.26

### Healthcare resource utilization and costs

The HCRU were consistently higher among mTNBC patients compared to Early and locally advanced TNBC [Table 2 (the assessment only considered the period when a therapy claim was reported, from the first claim for a breast-related therapy or surgery until the last claim for a breast-related therapy. The period between treatments is not included in the assessment)]. The overall costs for mTNBC management were 36% higher than that observed for patients with early and locally advanced disease, with a total PPPM cost of USD 10,005.95 [95% CI 9014.3–11,391.44] for metastatic and USD 7351.72 [95% CI 6692.27–8546.77] for early and locally advanced TNBC patients [Table 3 (the assessment only considered the period when a therapy claim was reported, from the first claim for a breast-related therapy or surgery until the last claim for a breast-related therapy. The period between treatments is not included in the assessment. Costs are presented in Brazilian Reais (BRL) and US dollar (USD))].

**Table 2:** HCRU for early and locally advanced and metastatic TNBC patients.

	Early TNBC	mTNBC
	N = 1034	N = 516
Inpatient setting		
Number of inpatient admissions		
PPPM [CI]	0.23 [0.20–0.27]	0.38 [0.32–0.44]
Mean ± SD	0.74 ± 1.31	1.69 ± 2.59
Length of stay in hospital days)		
Mean ± SD	1.44 ± 5.86	4.13 ± 10.07
Total days in hospital per patient		
Mean ± SD	2.70 ± 9.84	12.81 ± 30.54
Outpatient setting		
Number of ER visits		
PPPM	0.25 [0.22–0.29]	0.60 [0.51–0.72]
Mean ± SD	0.94 ± 2.52	2.76 ± 4.57
Number of outpatient visits		
PPPM	0.37 [0.32–0.41]	0.66 [0.58–0.74]
Mean ± SD	1.50 ± 4.00	3.98 ± 7.41
Number of procedures		
PPPM	23.15 [20.76–26.58]	34.60 [30.43–40.29]
Mean ± SD	77.97 ± 105.57	184.65 ± 206.74

**Table 3:** Costs related to early and locally advanced and metastatic TNBC management.

	Early and locally advanced TNBC		mTNBC	
	N = 1034		N = 516	
	BRL	USD	BRL	USD
Total costs				
PPPM [CI]	37,345.27 [33,995.4–43,415.87]	7351.72 [6692.27–8546.77]	50,828.22 [45,790.84–57,866.27]	10,005.95 [9014.3–11,391.44]
Median	87,154.75	17,157.12	151,772.79	29,877.7
IQI	50,927.22–13,2293.28	10,025.44–26,043.00	89,876.51–363,245.39	17,692.92–71,507.8
Inpatient costs				
PPPM [CI]	6912.21 [3785.94–12,934.26]	1360.72 [745.29–2546.21]	15,990.6 [11,046.48–23,096.16]	3147.88 [2174.59–4546.67]
Median	0	0	11,438.2	2251.7
IQI	0–11,848.42	0–2332.46	0–52,480.02	0–10,331.12
Outpatient costs				
PPPM [CI]	30,428.89 [29,296.72–31,609.1]	5990.17 [5767.3–6222.51]	34,808.16 [32,952.21–36,735.21]	6852.27 [6486.91–7231.62]
Median	80,144.3	15,777.05	127,916.86	25,181.47
IQI	47,330.78–123,046.58	9317.45–24,222.72	68,008.79–247,029.72	13,388.08–48,629.8

Among early and locally advanced TNBC patients, those undergoing NAT seemed to present the highest HCRU, due to ER visits, although comparing AT and NAT in this scenario has limitations to interpretation as the confidence intervals are broad and overlaps (Table 4). For mTNBC patients, HCRU gradually increases as the treatment line progresses. Early and locally advanced TNBC patients at a progressive stage presented an increased number of ER visits, procedures, and inpatient admissions reflecting the LOT1 for mTNBC patients.

**Table 4:** HCRU of early and locally advanced and metastatic TNBC patients, according to treatment setting.

	Number of ER visits		Number of procedures		Number of inpatient admissions	
	PPPM [CI]	Mean ± SD	PPPM [CI]	Mean ± SD	PPPM [CI]	Mean ± SD
Early and locally advanced TNBC						
NAT	0.35 [0.23–0.51]	1.10 ± 2.25	24.50 [21.02–28.81]	74.23 ± 105.23	0.28 [0.20–0.37]	0.90 ± 1.57
AT	0.24 [0.21–0.28]	0.71 ± 1.55	23.42 [20.32–27.91]	66.87 ± 88.32	0.23 [0.19–0.27]	0.61 ± 1.20
NAT/AT	0.24 [0.18–0.31]	1.34 ± 4.20	20.05 [18.16–22.19]	92.06 ± 97.16	0.21 [0.17–0.25]	0.88 ± 1.18
Progressive disease	0.57 [0.32–0.91]	2.13 ± 4.01	53.37 [32.44–91.83]	143.31 ± 130.44	0.48 [0.28–0.76]	1.24 ± 1.45
mTNBC						
NAT	0.34 [0.22–0.49]	0.81 ± 1.82	19.48 [16.72–22.89]	45.88 ± 96.73	0.20 [0.13–0.30]	0.43 ± 0.78
LOT1	0.58 [0.48–0.70]	1.71 ± 3.53	35.71 [30.82–42.05]	118.65 ± 167.35	0.41 [0.34–0.48]	1.08 ± 1.90
LOT2	0.68 [0.51–0.88]	1.44 ± 2.95	43.06 [33.98–54.74]	87.52 ± 100.71	0.44 [0.33–0.56]	0.80 ± 1.66
LOT3	0.87 [0.51–1.35]	1.75 ± 3.62	48.09 [34.73–64.85]	93.31 ± 87.53	0.57 [0.34–0.84]	0.88 ± 1.38

Among early and locally advanced TNBC patients, a slightly higher disease management cost was associated with NAT (USD 8093.56 [95% CI 6948.49–9372.4]). Patients with only AT presented a total cost of USD 7462.39 [6601.66–9028.81], and patients with NAT/AT presented a total cost of USD 6260.32 [5859.45–6682.78] [Table 5 (considered from the first claim for a systemic therapy or surgery until the last claim for a systemic therapy within the treatment setting)].

**Table 5:** Costs related to early and locally advanced and metastatic TNBC management, according to the treatment setting.

	Early and locally advanced TNBC				mTNBC			
	NAT	AT	NAT/AT	Progressive disease	NAT	LOT1	LOT2	LOT3
Total costs (BRL)								
BRL PPPM [CI]	41,113.68 [35,296.94–47,609.94]	37,907.44 [33,535.13–45,864.58]	31,801.18 [29,764.85–33,947.21]	54,803.67 [39,004.71–78,107.52]	28,885.36 [25,126.36–32,961.91]	52,553.46 [46,605.15–60,341.57]	52,147.89 [44,530.52–61,799.61]	48,349.43 [37,764.39–60,692.88]
BRL median	83,329.44	79,487.12	106,100.45	98,920.24	39,177.63	89,714.03	89,686.41	68,620.64
BRL IQI	48,831.52–139,243.14	46,109.98–124,199.4	75,336.07–163,217.68	51,518.80–194,940.75	17,493.07–53,673.66	40,314.26–199,409.62	52,278.58–130,561.48	29,334.41–129,150.62
Total costs (USD)								
USD PPPM [CI]	8093.56 [6948.49–9372.40]	7462.39 [6601.66–9028.81]	6260.32 [5859.45–6682.78]	10,788.55 [7678.39–15,376.10]	5686.32 [4946.33–6488.82]	10,345.57 [9174.60–11,878.73]	10,265.73 [8766.19–12,165.75]	9517.98 [7434.23–11,947.89]
USD median	16,404.08	15,647.68	20,886.73	19,473.25	7712.43	17,660.93	17,655.5	13,508.53
USD IQI	9612.88–27,411.14	9077.12–24,449.66	14,830.52–32,130.72	10,141.89–38,375.67	3443.65–10,566.09	7936.19–39,255.40	10,291.46–25,702.08	5774.72–25,424.35
Inpatient costs (BRL)								
BRL PPPM [CI]	5497.57 [2893.86–9168.40]	7780.03 [3635.05–15,775.53]	3131.80 [2362.86–4056.67]	21,545.15 [6882.90–45,306.83]	3692.12 [1975.69–5925.32]	17,316.44 [11,505.02–25,200.43]	20,740.76 [13,303.45–30,418.4]	18,839.79 [10,040.86–29,732.48]
BRL median	0	0	2125.20	8731.02	0	0	0	0
BRL IQI	0–12,750.24	0–9181.07	0–18,132.13	0–47,797.71	0–10,748.96	0–26,739.72	0–16,166.60	0–32,182.75
Inpatient costs (USD)								
USD PPPM [CI]	1082.24 [569.68–1804.87]	1531.56 [715.59–3105.54]	616.52 [465.15–798.59]	4241.34 [1354.95–8919.02]	726.82 [388.93–1166.45]	3408.88 [2264.86–4960.91]	4082.99 [2618.89–5988.11]	3708.77 [1976.62–5853.08]
USD median	0	0	418.36	1718.77	0	0	0	0
USD IQI	0–2509.99	0–1807.37	0–3569.46	0–9409.37	0–2116.02	0–5263.93	0–3182.53	0–6335.44
Outpatient costs (BRL)								
BRL PPPM [CI]	35,501.12 [30,766.87–40,636.14]	30,116.82 [28,762.15–31,537.38]	28,655.59 [26,934.02–30,450.09]	32,946.58 [25,966.11–40,592.17]	25,125.37 [21,516.59–29,090.01]	35,180.56 [32,838.35–37,643.78]	31,349.46 [28,740.28–34,196.80]	29,188.13 [22,105.57–38,526.77]
BRL median	77,558.13	73,559.47	98,373.43	67,164.56	32,487.18	66,362.23	69,190.96	38,052.96
BRL IQI	45,418.77–120,046.9	42,075.21–115,967.73	71,013.72–140,972.19	37,109.46–139,229.47	12,144.87–47,279.41	25,179.85–149,559.34	29,514.27–102,173.70	17,690.44–97,874.47
Outpatient costs (USD)								
USD PPPM [CI]	6988.68 [6056.71–7999.55]	5928.74 [5662.06–6208.39]	5641.09 [5302.18–5994.35]	6485.80 [5111.64–7990.90]	4946.13 [4235.72–5726.60]	6925.58 [6464.50–7410.48]	6171.40 [5657.76–6731.92]	5745.92 [4351.66–7584.31]
USD median	15,267.95	14,480.78	19,365.61	13,221.89	6395.36	13,063.94	13,620.80	7491.03
USD IQI	8941.05–23,632.20	8282.85–22,829.19	13,979.63–27,751.52	7305.3–27,408.45	2390.82–9307.33	4956.86–29,441.97	5810.12–20,113.72	3482.51–19,267.38

Costs are presented in Brazilian Reais (BRL) and US dollar (USD). Patients in a progressive stage presented a similar cost observed in metastatic patients, with a total expenditure of USD 10,788.55 [95% CI 7678.39–15,376.10]. In mTNBC patients, the costs were lower for patients undergoing more advanced LOT, with a total cost of USD 9517.98 [95% CI 7434.23–11,947.89] for LOT3, USD 10,265.73 [95% CI 8766.19–12,165.75] for LOT2 and USD 10,345.57 [95% CI 9174.6–11,878.73] for LOT1. Although, it is worth noticing that the smaller number of patients in LOT3 may have impacted this analysis, and that the confidence intervals of LOT1, LOT2 and LOT3 overlaps.

**Figure 3:**
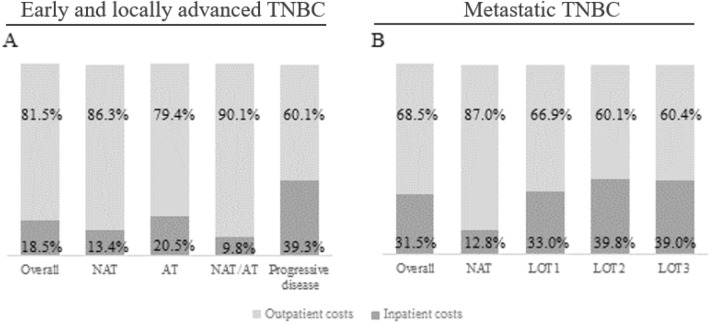
Relative costs associated with early and locally advanced (**A**) and metastatic (**B**) TNBC management.

The outpatient setting was the major contributor for TNBC management expenditures, representing 81.5% and 68.5% of the total costs for early and locally advanced and metastatic TNBC patients, respectively (Fig. 3).

## Discussion

The Brazilian health system comprises a public and a private sector. The public one, Sistema Único de Saúde (SUS, Portuguese to Unified Health System) is state founded by the thought Ministry of Health and available for all Brazilian citizens, but 75% of the population relies only at SUS to have access to health care. On the other hand, around 25% of the population has supplementary health care at the private sector, which is linked to care through individual or family contracts (19%), business (68%) and collective (13%)^25,26^. To our knowledge, this is the first study to describe the treatment patterns, HCRU and costs associated with the management of Brazilian TNBC patients from the perspective of the private healthcare setting. As expected, TNBC patients comprised a small part of all BC patients identified in the Orizon database, and the majority was diagnosed at an early and locally advanced stage. For early and locally advanced TNBC, over 75% of patients received AT, mainly with anthracycline followed by taxane-based regimens. In the neoadjuvant setting, the combination of anthracycline and taxane was also the chemotherapy of choice. The preferred treatment for metastatic patients were bevacizumab-based regimens. A greater use of resources and more expensive management was seen for mTNBC patients; among early and locally advanced TNBC patients’ NAT was associated with a greater use of resources and higher treatment costs.

Previous observational studies conducted in Brazil with data obtained from medical records have demonstrated that the TNBC subtype accounts for 15.6% to 21% of all BC^4,12,27,28^. Here, we observed a lower proportion (6%). Missing ICD-10 information, and the lack of clinical data may have contributed to the loss of some TNBC patients in the database. Also, the number of BC patients may be overestimated, since some patients may have had ICD-10 C50 reported for procedures performed for screening purposes only, without actual confirmation of the disease.

We observed that TNBC patients were distributed mainly throughout the Southeastern region. Simon et al. previously showed that, regardless of the BC subtypes, most patients were from the Southeastern region, even though the North and Northeastern regions presented a high proportion of patients with TNBC^4^. Here, a selection bias could have contributed to this distribution as the Southeastern region presents the highest rates of private health insurance coverage. The identification of a breast-related surgery was a key point to classify the therapy scheme prescribed, however, the proportion of early and locally advanced TNBC patients with a documented surgery was much less (41.6%) than expected (85–90%)^29,30^, while the rate of surgery was higher than expected in the metastatic setting (37.4%). We assume that the lower proportion of surgery in early stage was related to: (i) surgical procedures with an exclusive HMO codification which hindered the algorithm search; or (ii) censored data. Furthermore, there are some reports that could help in clarifying the reason for the discrepant rate of surgery for the metastatic disease: an American study with over 24,000 women showed that de novo metastatic breast cancer had surgery in over 40% of the cases^31^. At our database it is not possible to know how many of them had stage IV at diagnosis. A Brazilian recent analysis with a smaller number of cases, around 10% of breast cancer had surgery to metastatic disease management, with a tendency of increased proportion over the years (4.5% in 1995–2003 to 12.5% in 2004–2011)^32^, although this study period is before our database.

Nevertheless, for a comprehensive assessment of treatment patterns and HCRU, we only considered the subgroup of early and locally advanced TNBC patients who underwent a breast-related surgery. It is worth noticing as well that some procedures were included as package codes for which definitions weren’t available, this database limitation could have resulted in the underrepresentation of radiation therapy. In Brazil several studies had reported higher rates of radiation therapy in the curative setting, reaching 85%^33,34^.

In general, BC patients who receive chemotherapy in Brazil are usually prescribed with anthracycline and taxane-based regimens^30^, which was also observed in this study, in both combined and sequential regimes. Notably, platinum regimens were rarely observed, as their use for TNBC management remains controversial. Although several studies indicate that the addition of platinum drugs to the neoadjuvant regimens could increase pathological complete response^35,36^, few patients were treated with such drugs during the time range evaluated in this study period (2014–2017). For patients receiving NAT, Silva et al. reported that the most common regimens used are AC-T and FAC-T (fluorouracil plus doxorubicin plus cyclophosphamide followed by docetaxel)^13^. Herein, we also observed AC-T as the main combination regimen in the neoadjuvant setting. For metastatic patients, bevacizumab, gemcitabine, and capecitabine were the main agents used, alone or in combination, which was very similar to what was reported in other countries^37,38^.

Silva et al. reported that in the public setting the median time from diagnosis until NAT initiation was 3 months^13^. In the private setting, we observed a shorter period, with an average of 0.3 months until the beginning of NAT and 0.7 months until the breast-related surgery. As stated by Silva, the delay in treatment initiation could have a negative impact on clinical outcomes, but despite the very effective treatment initiation observed, we assumed that 4.4% of early and locally advanced patients did progress during the study period.

Metastatic disease was associated with high HCRU and costs, which is in accordance with other real-world studies conducted worldwide^37,39^. However, in other countries, a considerable difference in total costs was seen based on the stage of the disease. In a study conducted in Ontario, Brezden et al. reported that for stage I-III TNBC patients, average annual per-patient costs were $ 35,064 and for stage IV patients the costs were $ 140,160^37^. In the US, Schwartz et al. reported that the mean PPPM costs were $4810 for patients with stage III and $9159 for patients with stage IV TNBC^39^. In both cases, the total costs were mainly associated with ambulatory and inpatient care and comprised the full period of care. Herein, the cost differences between early and locally advanced and metastatic management were not so pronounced, since we accounted only for the period while the patients were under a breast cancer-related chemotherapy. Nevertheless, the higher expenditure for mTNBC patients was associated with the inpatient setting costs.

In the present study we also observed that for metastatic patients HCRU increased as the treatment line advanced, although the increase is not substantial. This small difference in costs can be explained by the different pattern of regimens used between treatment lines, and the shorter duration of treatments as the lines progress. Early and locally advanced patients with progressive disease presented similar HCRU pattern and treatment costs as metastatic patients. Early and locally advanced TNBC patients treated with NAT had a higher number of ER visits and inpatient admissions. Since NAT is used most often for locally advanced breast cancer patients to convert unresectable tumors into resectable ones^8^, it is expected that the management for these patients would be more complex, leading to a more frequently inpatient and ER admissions.

This study cohort reports the treatment patterns and HRCU for TNBC from 2014 to 2017, therefore it doesn’t include the new technologies that were approved into the disease landscape, for instance: the PARP inhibitors were approved in 2019 and 2022 (for advanced and early stage gBRCAm HER2- Breast Cancer, respectively) but, still there is restriction to its use in either setting because an additional HTA assessment is required for oral drugs incorporation into private health sector^25,26,40,41^. In the other hand, immune check-point inhibitors (ICI) were approved in 2019 and 2022 (atezolizumab for metastatic TNBC and pembrolizumab for early and metastatic TNBC, respectively)^42,43^. Additionally, in 2022 were also approved antibody–drug conjugates trastuzumab deruxtecan for HER2-low^44^ and the sacituzumab govitecan^45^, both in latter lines of metastatic TNBC. Nonetheless, the scenario depicted in this study still represents the reality of public health sector in Brazil where none of these technologies were incorporated since their approvals, as it requires health technology assessment by CONITEC, a committee from the Ministry of Health^46^. Due to this anachronical timeframes, it is expected that a new study assessing the current private health sector treatment patterns and HCRU may result in a different portrait, with higher HCRU in both early and advanced stages of TNBC. However, as both PARP inhibitors and ICI were approved for NAT/AT curative TNBC and had been previously reported as cost-effective abroad^47–49^, it is possible to infer that treating early stage TNBC remains leading to a lower HCRU. To confirm this information, new studies should be conducted.

Limitations of our study include those inherent to administrative claims database analysis, specially, missing clinical variables and underreported data. Additionally, the period that each patient has available data in the database may not assess all relapses occurrences. Also, it was not possible to assess comprehensively the cost of medication in the database nor the reasons for hospital readmission. The assumptions used to identify TNBC patients may have resulted in a selection bias, as the ICD-10 code is not a required field in the Orizon database. In addition, the staging classification relied on the treatment and report of any claim related to metastatic disease management. As most of the cytotoxic agents used in the (neo)adjuvant setting is often used as frontline therapy for mTNBC, some patients with metastatic disease could have been misclassified. Also, some patients with early and locally advanced TNBC at index date may have been classified as metastatic if they were treated with drugs used for metastasis management within 6 months of the index date. In addition, some procedures and medications were included as package codes whose definition was not available, this could have potentially impact on the radiation therapy in AT. This may have compromised the identification of patients who underwent breast surgery and hindered the medication cost assessment. Censored data was not possible to address. Patients could have withdrawn from the database due to death, loss of coverage, or end of treatment.

## Conclusion

In conclusion, our study demonstrated that anthracycline and taxane-based regimens given as adjuvant therapy were the mainstay treatments for early and locally advanced TNBC patients in the private healthcare setting. For metastatic patients, bevacizumab, gemcitabine, and capecitabine-based regimens were often prescribed. Although the proportion of patients receiving a subsequent systemic treatment decreased up to 12% in LOT3, in terms of overall costs treating mTNBC presented a higher HCRU compared to the curative setting. This data here presented reinforces the need to increase cure rates as a pathway to optimize resources allocation in breast cancer management.

### Supplementary Information

Supplementary Tables.

## Data Availability

The data that support the findings of this study are available from IQVIA with restrictions since the data were used under license for the current study, and so are not publicly available. Data are however available from the authors upon reasonable request and with permission of IQVIA.

## References

[1] Bauer KR, Brown M, Cress RD, Parise CA, Caggiano V (2007). Descriptive analysis of estrogen receptor (ER)-negative, progesterone receptor (PR)-negative, and HER2-negative invasive breast cancer, the so-called triple-negative phenotype: A population-based study from the California cancer Registry. Cancer.

[2] American Cancer Society. Cancer facts and figures 2018 (2018).

[3] Dent R, Trudeau M, Pritchard KI, Hanna WM, Kahn HK, Sawka CA (2007). Triple-negative breast cancer: Clinical features and patterns of recurrence. Clin. Cancer Res..

[4] Simon SD (2019). Characteristics and prognosis of stage I–III breast cancer subtypes in Brazil: The AMAZONA retrospective cohort study. Breast.

[5] Furlanetto J, Loibl S (2020). Optimal systemic treatment for early triple-negative breast cancer. Breast Care.

[6] Diana A, Carlino F, Franzese E, Oikonomidou O, Criscitiello C, De Vita F (2020). Early triple negative breast cancer: Conventional treatment and emerging therapeutic landscapes. Cancers.

[7] Gupta GK, Collier AL, Lee D, Hoefer RA, Zheleva V, van Reesema LLS (2020). Perspectives on triple-negative breast cancer: Current treatment strategies, unmet needs, and potential targets for future therapies. Cancers.

[8] Tufano AM, Teplinsky E, Landry CA (2020). Updates in neoadjuvant therapy for triple negative breast cancer. Clin. Breast Cancer.

[9] Mauri D, Pavlidis N, Ioannidis JPA (2005). Neoadjuvant versus adjuvant systemic treatment in breast cancer: A meta-analysis. J. Natl. Cancer Inst..

[10] Medina MA, Oza G, Sharma A, Arriaga LG, Hernández JMH, Rotello VM (2020). Triple-negative breast cancer: A review of conventional and advanced therapeutic strategies. Int. J. Environ. Res. Public Health.

[11] Kaufmann M, Hortobagyi GN, Goldhirsch A, Scholl S, Makris A, Valagussa P (2006). Recommendations from an international expert panel on the use of neoadjuvant (primary) systemic treatment of operable breast cancer: An update. J. Clin. Oncol..

[12] Gonçalves H, Guerra MR, Rocha J, Cintra D, Fayer VA, Brum IV (2018). Survival study of triple-negative and non-triple-negative breast cancer in a Brazilian cohort. Clin. Med. Insights Oncol..

[13] da Silva JL, de Paula BHR, Small IA, Thuler LCS, de Melo AC (2020). Sociodemographic, clinical, and pathological factors influencing outcomes in locally advanced triple negative breast cancer: A Brazilian cohort. Breast Cancer Basic Clin. Res..

[14] Rala de Paula BH, Kumar S, Morosini FM, Calábria Cardoso DEM, MoreiradeSousa CA, Crocamo S (2020). Real-world assessment of the effect of impact of tumor size on pathological complete response rates in triple negative breast cancer after neoadjuvant chemotherapy. Chin. Clin. Oncol..

[15] Baser O, Wei W, Henk HJ, Teitelbaum A, Xie L (2012). Patient survival and healthcare utilization costs after diagnosis of triple-negative breast cancer in a United States managed care cancer registry. Curr. Med. Res. Opin..

[16] Schwartz KL, Simon MS, Bylsma LC (2018). Clinical and economic burden associated with stage III to IV triple-negative breast cancer: A SEER-Medicare historical cohort study in elderly women in the United States. Cancer.

[17] Aly A, Shah R, Hill K, Botteman MF (2019). Overall survival, costs and healthcare resource use by number of regimens received in elderly patients with newly diagnosed metastatic triple-negative breast cancer. Futur. Oncol..

[18] Hirai, S. *et al.* Câncer no Brasil: A jornada do paciente no sistema de saúde e seus impactos sociais e financeiros (2019).

[19] Ades F (2017). Access to oncology drugs in Brazil: Juggling innovation and sustainability in developing countries. Med. Access. @ Point Care.

[20] ANS (Agência Nacional de Saúde Suplementar). ANS TabNet-Informações em Saúde Suplementar. https://www.ans.gov.br/anstabnet/cgi-bin/tabnet?dados/tabnet_br.def (Accessed 02 Sept 2023).

[21] Edge SB, Compton CC (2010). The American Joint Committee on Cancer: The 7th edition of the AJCC cancer staging manual and the future of TNM. Ann. Surg. Oncol..

[22] Blumen H, Fitch K, Polkus V (2016). Comparison of treatment costs for breast cancer, by tumor stage and type of service. Am. Health Drug Benefits.

[23] Nordstrom BL, Whyte JL, Stolar M, Mercaldi C, Kallich JD (2012). Identification of metastatic cancer in claims data. Pharmacoepidemiol. Drug Saf..

[24] Meng W, Ou W, Chandwani S, Chen X, Black W, Cai Z (2019). Temporal phenotyping by mining healthcare data to derive lines of therapy for cancer. J. Biomed. Inform..

[25] Cruz JAW, da Cunha M, de Moraes TP (2022). Brazilian private health system: History, scenarios, and trends. BMC Health Serv. Res..

[26] Agência Nacional de Saúde Suplementar. Dados e Indicadores do Setor (2023) https://www.gov.br/ans/pt-br/acesso-a-informacao/perfil-do-setor/dados-e-indicadores-do-setor (Accessed 02 Sept 2023).

[27] de Macêdo Andrade AC, Ferreira Júnior CA, Dantas Guimarães B, Pessoa Barros AW, de Almeida GS, Weller M (2014). Molecular breast cancer subtypes and therapies in a public hospital of Northeastern Brazil. BMC Womens Health.

[28] Rosa DD, Bines J, Werutsky G, Barrios CH, Cronemberger E, Queiroz GS (2020). The impact of sociodemographic factors and health insurance coverage in the diagnosis and clinicopathological characteristics of breast cancer in Brazil: AMAZONA III study (GBECAM 0115). Breast Cancer Res. Treat..

[29] Boukai A, Gonçalves AC, Padoan M, Andrade P, Carvalho N, Lemos F (2018). Outcome of patients with breast cancer treated in a private health care institution in Brazil original report. J. Glob. Oncol..

[30] Liedke PER, Finkelstein DM, Szymonifka J, Barrios CH, Chavarri-Guerra Y, Bines J (2014). Outcomes of breast cancer in Brazil related to health care coverage: A retrospective cohort study. Cancer Epidemiol. Biomark. Prev..

[31] Lane WO, Thomas SM, Blitzblau RC, Plichta JK, Rosenberger LH, Fayanju OM, Hyslop T, Hwang ES, Greenup RA (2019). Surgical resection of the primary tumor in women with de novo stage IV breast cancer: Contemporary practice patterns and survival analysis. Ann. Surg..

[32] Soares LR, Freitas-Junior R, Nunes RD, Martins E, Oliveira JC, Curado MP (2022). Real-world data on metastatic breast cancer in Goiânia, Brazil: A 17-year analysis (1995–2011). Mastology.

[33] Andrade DAP, Veneziani AC, Paiva CE, Reis R, Filho CAF, Sanches AON (2023). Discrepancies in breast cancer’s oncological outcomes between public and private institutions in the southeast region of Brazil: A retrospective cohort study. Front. Oncol..

[34] Marta GN, AlBeesh R, Pereira AAL, Oliveira LJ, Mano MS, Hijal T (2020). The impact on clinical outcomes of post-operative radiation therapy delay after neoadjuvant chemotherapy in patients with breast cancer: A multicentric international study. Breast.

[35] Pandy JGP, Balolong-Garcia JC, Cruz-Ordinario MVB, Que FVF (2018). Triple negative breast cancer and platinum-based systemic treatment: Meta-analysis and systematic review. Ann. Oncol..

[36] Poggio F, Bruzzone M, Ceppi M, Pondé NF, La Valle G, Del Mastro L (2018). Platinum-based neoadjuvant chemotherapy in triple-negative breast cancer: A systematic review and meta-analysis. Ann. Oncol..

[37] Brezden-Masley C, Fathers KE, Coombes ME, Pourmirza B, Xue C, Jerzak KJ (2020). A population-based comparison of treatment patterns, resource utilization, and costs by cancer stage for Ontario patients with triple-negative breast cancer. Cancer Med..

[38] Schilling J, Busch S, Heinrich G, Heinig K, Martin Kurbacher C, Klare P (2019). Treatment patterns, health care resource use and outcomes in metastatic triple-negative breast cancer in Germany: Retrospective chart review study (OBTAIN). Curr. Med. Res. Opin..

[39] Schwartz KL, Simon MS, Bylsma LC, Ruterbusch JJ, Beebe-Dimmer JL, Schultz NM (2018). Clinical and economic burden associated with stage III to IV triple-negative breast cancer: A SEER-Medicare historical cohort study in elderly women in the United States. Cancer.

[40] LYNPARZA: Comprimidos. Responsável técnico Mauricio Rivas Marante (AstraZeneca do Brasil Ltda, 2023). https://consultas.anvisa.gov.br/#/bulario/q/?numeroRegistro=116180268 (Accessed 2 Sept 2023).

[41] TALZENNA: Cápsulas Duras. Responsável técnico Andrea T. Nichele (Pfizer Brasil Ltda., 2023) https://consultas.anvisa.gov.br/#/bulario/q/?numeroRegistro=121100482 (Accessed 2 Sept 2023).

[42] KEYTRUDA: Solução injetável. Responsável técnico Fernando C. Lemos (Merck Sharp & Dohme Farmacêutica Ltda., 2023). https://consultas.anvisa.gov.br/#/bulario/q/?numeroRegistro=101710209 (Accessed 2 Sept 2023).

[43] TECENTRIQ: Solução para diluição para infusão ÍNDICE 840 mg. Responsável técnico Liana Gomes de Oliveira (Produtos Roche Químicos e Farmacêuticos S.A., 2023). https://consultas.anvisa.gov.br/#/bulario/q/?numeroRegistro=101000665 (Accessed 2 Sept 2023).

[44] ENHERTU: Pó liofilizado para solução injetável. Responsável técnico Pedro de Freitas Fiorante (Daiichi Sankyo Brasil Farmacêutica Ltda., 2023) https://consultas.anvisa.gov.br/#/bulario/q/?numeroRegistro=104540191 (Accessed 2 Sept 2023).

[45] TRODELVY: Pó liofilizado para solução injetável. Responsável técnico Denise Sunagawa (Gilead Sciences Farmacêutica do Brasil Ltda, 2023) https://consultas.anvisa.gov.br/#/bulario/q/?numeroRegistro=109290012 (Accessed 2 Sept 2023).

[46] Campolina AG, Yuba TY, Soárez PC (2022). Decision criteria for resource allocation: An analysis of CONITEC oncology reports. Cien. Saude Colet..

[47] Huang M, Fasching PA, Haiderali A, Xue W, Yang C, Pan W (2023). Cost-effectiveness of neoadjuvant pembrolizumab plus chemotherapy followed by adjuvant single-agent pembrolizumab for high-risk early-stage triple-negative breast cancer in the United States. Adv. Ther..

[48] Fasching PA, Huang M, Haiderali A, Xue W, Pan W, Zhou Z-Y (2022). Cost effectiveness of pembrolizumab in combination with chemotherapy as neoadjuvant therapy and continued as a single agent as adjuvant therapy for high-risk early-stage TNBC in the United States. Ann. Oncol..

[49] Zettler C, Silva D, Blinder VS, Robson ME, Elkin EB (2022). Cost effectiveness of adjuvant olaparib for BRCA-mutated, early-stage breast cancer. J. Clin. Oncol..

