# Supplementary material for: Treatment patterns and healthcare resource utilization for triple negative breast cancer in the Brazilian private healthcare system: a database study
Source: Sci Rep. 2023 Sep 22;13:15785. doi: 10.1038/s41598-023-43131-9 (PMC10516856; doi:10.1038/s41598-023-43131-9)
Supplement: Supplementary file 1 — Supplementary Tables. [file 41598_2023_43131_MOESM1_ESM.docx]

**Supplementary Information**

Supplementary table 1. List of inclusion and exclusion molecules used to identify patients with TNBC in Orizon database

| Inclusion criteria molecules | Treatments used for triple negative breast cancer:  Gemcitabine, irinotecan, irinotecan hydrochloride, gemcitabine hydrochloride, vinorelbine ditartrate, oxaliplatin, eribulin mesylate, cisplatin, capecitabine, paclitaxel, doxorubicin hydrochloride, docetaxel, carboplatin, bevacizumab, or fluorouracil. |
| --- | --- |
| Exclusion criteria molecules | Treatments used for HER+ and/or HR+ breast cancers:  Fulvestrant, tamoxifen citrate, anastrozole, letrozole, trastuzumab emtasine, trastuzumab or pertuzumab. |

Supplementary table 2. List of metastatic-related treatments and procedures

| Metastatic systemic agents for TNBC | Capecitabine, gemcitabine, liposomal doxorubicin, bevacizumab, eribulin, and vinorelbine. |
| --- | --- |
| Procedures related to metastasis management | Radiotherapy bone metastasis/myeloma  Radiotherapy of bone metastasis/myeloma  Teletherapy bone metastasis /myeloma  Radiotherapy - simple bone metastasis  Radiotherapy simple bone metastasis Bradesco  Radio bone metastasis  Radiotherapy - bone metastasis  Package (HP+HM) teletherapy brain metastasis  Enucleation of hepatic metastasis  Package radiotherapy total skull (inc brain metastasis) conv |

*Supplementary Table 3: Demographic information of TNBC patients according to tumor stage*

|  | **Early TNBC** | **mTNBC** | **All patients** |
| --- | --- | --- | --- |
| Total patients, n (%) | 2,488 (82.8) | 516 (17.2) | 3,004 (100.0) |
|  |  |  |  |
| Age at first claim |  |  |  |
| Mean ± SD | 49.61±12.62 | 48.43±12.54 | 49.41±12.62 |
| Median | 48 | 48 | 48 |
| Min - max | 19 - 91 | 20 - 90 | 19 – 91 |
| IQI | 40.00-58.00 | 38.00-57.00 | 40.00-58.00 |
|  |  |  |  |
| Proportion of patients according to age at first claim, n (%) | | | |
| 18-29 | 70 (2.8) | 12 (2.3) | 82 (2.7) |
| 30-39 | 262 (10.5) | 62 (12.0) | 324 (10.8) |
| 40-49 | 390 (15.7) | 76 (14.7) | 466 (15.5) |
| 50-59 | 314 (12.6) | 64 (12.4) | 378 (12.6) |
| 60-69 | 185 (7.4) | 35 (6.8) | 220 (7.3) |
| 70-79 | 66 (2.7) | 10 (1.9) | 76 (2.5) |
| ≥ 80 | 12 (0.5) | 2 (0.4) | 14 (0.5) |
| Missing information | 1189 (47.8) | 255 (49.4) | 1444 (48.1) |
|  |  |  |  |
| Follow-up (years) |  |  |  |
| Mean ± SD | 2.66±1.67 | 2.24±1.45 | 2.59±1.64 |
| Median | 2.25 | 1.91 | 2.18 |
| Min - max | 0.5 - 6.97 | 0.51 - 6.69 | 0.5 - 6.97 |
| IQI | 1.23-3.88 | 1.03-3.09 | 1.19-3.75 |
|  |  |  |  |
| Proportion of patients per Brazilian region, n (%) | |  |  |
| North | 39 (1.6) | 5 (1.0) | 44 (1.5) |
| Northeast | 475 (19.1) | 46 (8.9) | 521 (17.3) |
| Midwest | 180 (7.2) | 27 (5.2) | 207 (6.9) |
| Southeast | 1632 (65.6) | 425 (82.4) | 2057 (68.5) |
| South | 161 (6.5) | 13 (2.5) | 174 (5.8) |
|  |  |  |  |
| Treatment |  |  |  |
| Surgery, n (%) | 1,034 (41.6) | 193 (37.4) | 1,227 (40.8) |
| Radiotherapy, n (%) | 135 (5.4) | 42 (8.1) | 177 (5.9) |

*Supplementary Table 4. Regimens prescribed for early TNBC, according to treatment setting*

|  | **Early TNBC** | | | | | | |
| --- | --- | --- | --- | --- | --- | --- | --- |
|  | N=1,034 | | | | | | |
| **Regimens** | **NAT** | **AT** | | **NAT/AT** | | | **Progressive disease** |
|  | n= 78 | **AT** | **Sequential** | **NAT** | **AT** | **Sequential** | n=45 |
|  |  | n=779 | n=446 | n=177 | n=177 | n=23 |  |
| n (%) |  |  |  |  |  |  |  |
| Anthracycline-based | 10 (12.8) | 383 (49.2) | 43 (9.6) | 130 (73.4) | 24 (13.6) | 0 (0.0) | 4 (8.9) |
| Taxane-based | 25 (32.1) | 248 (31.8) | 350 (78.5) | 24 (13.6) | 123 (69.5) | 4 (4.4) | 4 (8.9) |
| Platinum-based | 0 (0.0) | 3 (0.4) | 1 (0.2) | 0 (0.0) | 1 (0.6) | 0 (0.0) | 0 (0.0) |
| Taxane+platinum-based | 4 (5.1) | 13 (1.7) | 6 (1.3) | 10 (5.6) | 12 (6.8) | 2 (2.2) | 2 (4.4) |
| Anthracycline+taxane-based | 36 (46.2) | 20 (2.6) | 10 (2.2) | 5 (2.8) | 5 (2.8) | 0 (0.0) | 0 (0.0) |
| Bevacizumab-based | 0 (0.0) | 12 (1.5) | 5 (1.1) | 0 (0.0) | 3 (1.7) | 8 (8.8) | 11 (24.4) |
| Capecitabine-based | 0 (0.0) | 10 (1.3) | 5 (1.1) | 0 (0.0) | 2 (1.1) | 4 (4.4) | 9 (20.0) |
| Gemcitabine-based | 1 (1.3) | 17 (2.2) | 15 (3.4) | 3 (1.7) | 1 (0.6) | 3 (3.3) | 9 (20.0) |
| Other | 2 (2.6) | 73 (9.4) | 11 (2.5) | 5 (2.8) | 6 (3.4) | 2 (2.2) | 6 (13.3) |

*Supplementary Table 5. Regimens prescribed for mTNBC, according to line of treatment*

|  | **mTNBC** | | | |
| --- | --- | --- | --- | --- |
|  | N=516 | | | |
|  | **NAT** | **LOT1** | **LOT2** | **LOT3** |
|  | n=77 | n=509 | n=250 | n=65 |
|  |  |  |  |  |
| Anthracycline-based | 58 (74.4) | 97 (19.1) | 29 (11.6) | 6 (9.2) |
| Taxane-based | 8 (10.3) | 101 (19.8) | 86 (34.4) | 5 (7.7) |
| Platinum-based | 0 (0) | 22 (4.3) | 7 (2.8) | 1 (1.5) |
| Taxane+platinum-based | 3 (3.8) | 55 (10.8) | 13 (5.2) | 2 (3.1) |
| Anthracycline+taxane-based | 3 (3.8) | 3 (0.6) | 1 (0.4) | 0 (0) |
| Bevacizumab-based | 1 (1.3) | 110 (21.6) | 21 (8.4) | 5 (7.7) |
| Capecitabine-based | 2 (2.6) | 12 (2.4) | 16 (6.4) | 14 (21.5) |
| Gemcitabine-based | 1 (1.3) | 72 (14.1) | 46 (18.4) | 10 (15.4) |
| Other | 1 (1.3) | 37 (7.3) | 31 (12.4) | 22 (33.8) |
